# Supplementary figures and images for: Prospective evaluation of deep learning image reconstruction for Lung-RADS and automatic nodule volumetry on ultralow-dose chest CT
Source: PLoS One. 2024 Feb 22;19(2):e0297390. doi: 10.1371/journal.pone.0297390 (PMC10883577; doi:10.1371/journal.pone.0297390)

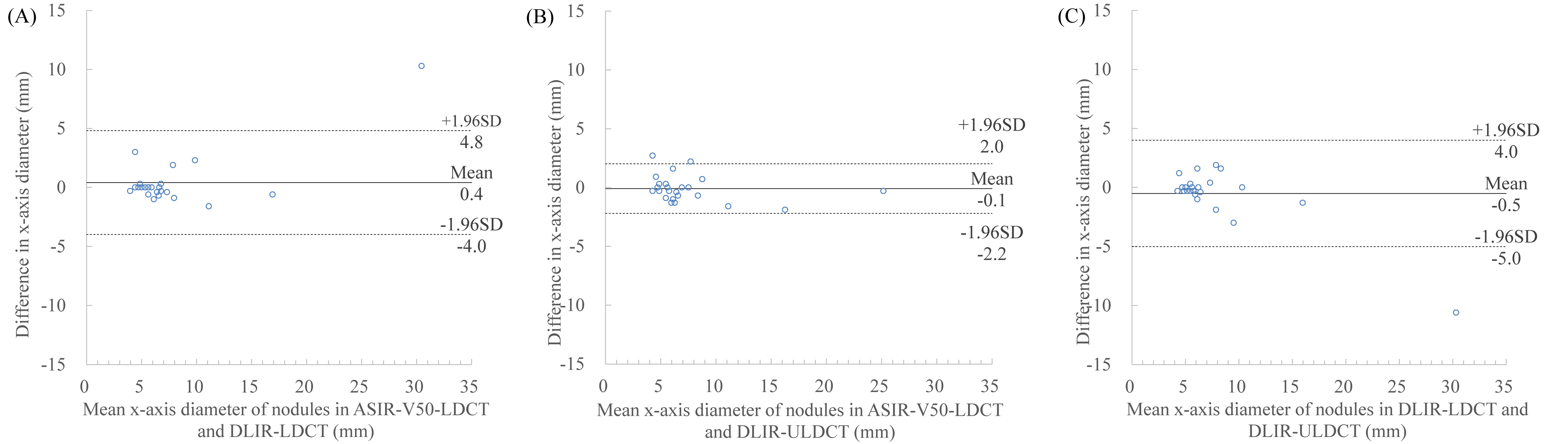

Supplement: S1 Fig — Compared to low-dose chest CT with adaptive statistical iterative reconstruction-V 50% (ASIR-V50), low-dose chest CT with deep-learning image reconstruction (DLIR) and ultralow-dose chest CT with DLIR showed mean x-axis diameter differences of 0.4 mm (-4.0, 4.8) (a) and -0.1 mm (-2.2, 2.0), respectively (b). In terms of deep learning image reconstruction, low-dose chest CT and ultralow-dose chest CT showed a mean x-axis diameter difference of -0.5 mm (-5.0, 4.0) (c). LDCT = low-dose chest computed tomography, ULDCT = ultralow-dose chest computed tomography, ASIR-V50 = adaptive statistical iterative reconstruction-V 50%, DLIR = deep-learning image reconstruction, VCAR = volume computerized assisted reporting. (TIF) [file pone.0297390.s001.tif]

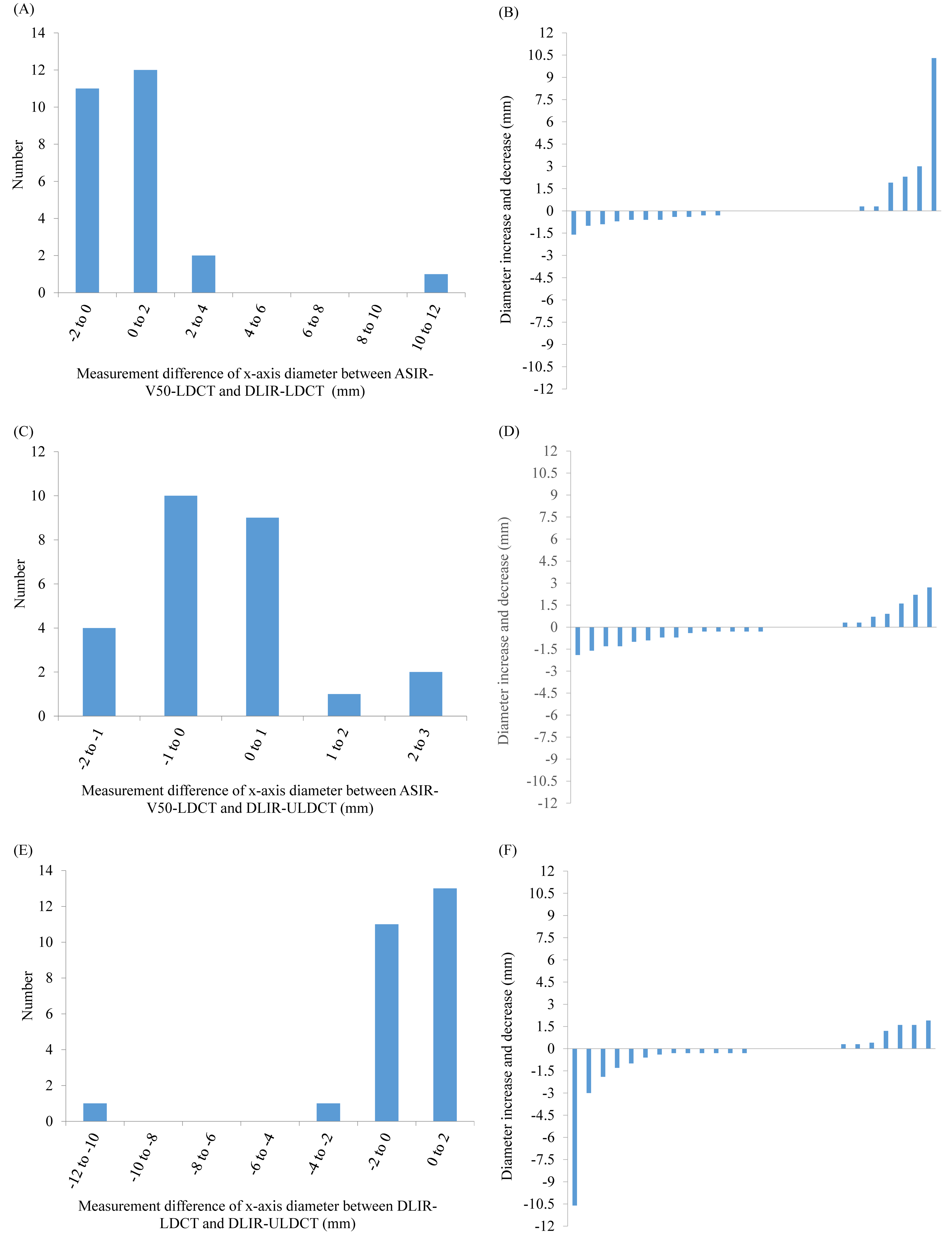

Supplement: S2 Fig — Distribution of measurement differences between low-dose chest CT with adaptive statistical iterative reconstruction-V 50% (ASIR-V50) and low-dose chest CT with deep-learning image reconstruction (DLIR) (a,b), low-dose chest CT with ASIR-V50 and ultralow-dose chest CT with DLIR (c, d), and low-dose chest CT and ultralow-dose chest CT with DLIR-H (e, f). In total, 76.9–80.7% of the nodules showed a measurement difference of the diameter equal to or less than 1.5 mm. LDCT = low-dose chest computed tomography, ULDCT = ultralow-dose chest computed tomography, ASIR-V50 = adaptive statistical iterative reconstruction-V 50%, DLIR = deep-learning image reconstruction. (TIF) [file pone.0297390.s002.tif]

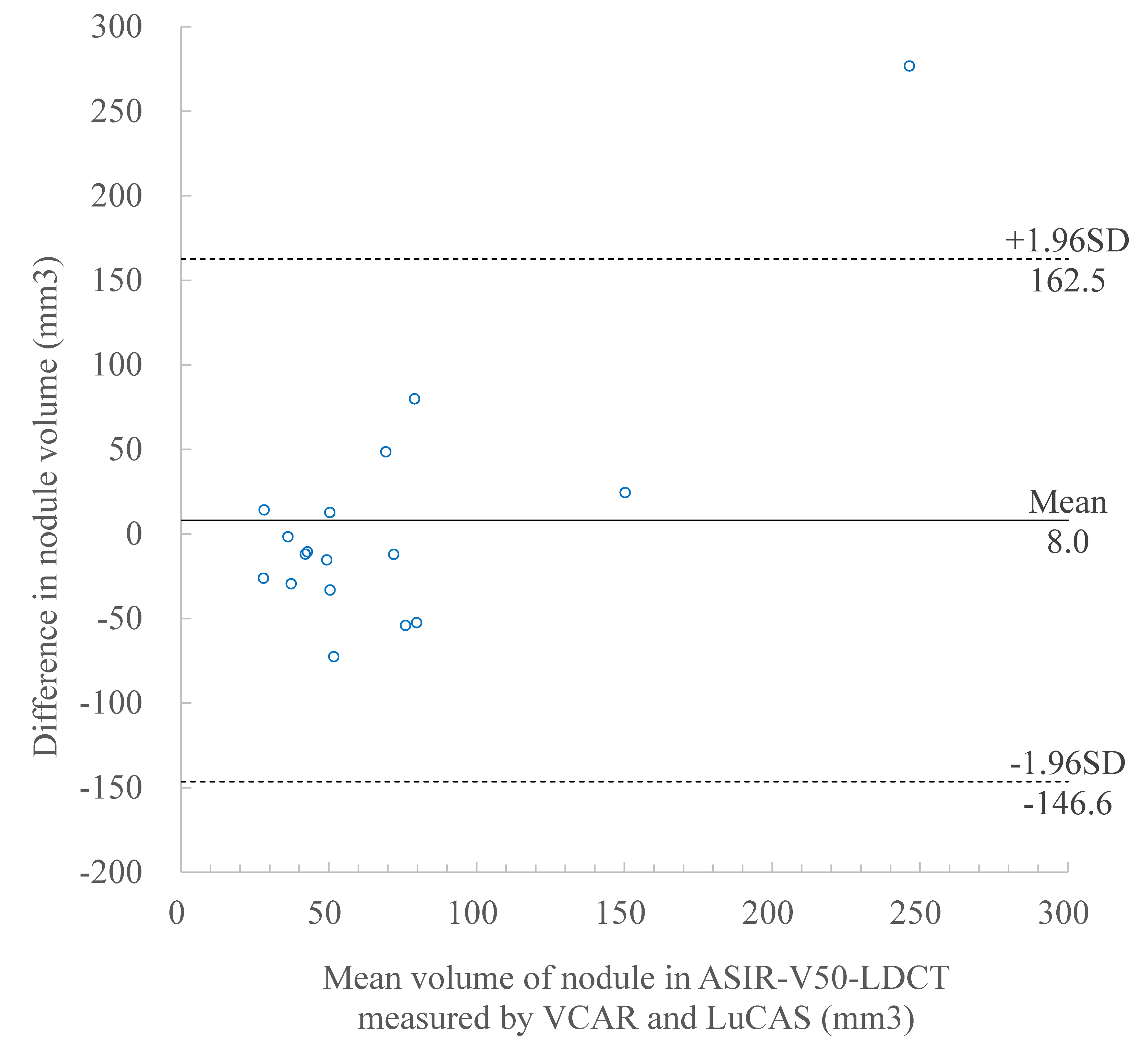

Supplement: S3 Fig — ASIR-V50 = adaptive statistical iterative reconstruction-V 50%, LDCT = low-dose chest computed tomography, VCAR = volume computerized assisted reporting. (TIF) [file pone.0297390.s003.tif]
