# Supplementary material for: Prospective evaluation of deep learning image reconstruction for Lung-RADS and automatic nodule volumetry on ultralow-dose chest CT
Source: PLoS One. 2024 Feb 22;19(2):e0297390. doi: 10.1371/journal.pone.0297390 (PMC10883577; doi:10.1371/journal.pone.0297390)
Supplement: S1 Table — (DOCX) [file pone.0297390.s004.docx]

**S1 Table. Scoring System of Subjective Image Quality**

| **Score** | **Image noise** | **Visibility of small structures** | **Lesion conspicuity** | **Diagnostic acceptability for clinical interpretation** |
| --- | --- | --- | --- | --- |
| 5 | Minimal | Excellent visualization | Well-seen lesion with well-visualized margins |  |
| 4 | Less than average | Better than average | Better than average | Fully acceptable |
| 3 | Average | Average | Average | Probably acceptable |
| 2 | More than average | Less than average | Less than average | Acceptable only in limited condition |
| 1 | Unacceptable | Unacceptable | Cannot identify | Diagnostically unacceptable |
