# Supplementary material for: Prospective evaluation of deep learning image reconstruction for Lung-RADS and automatic nodule volumetry on ultralow-dose chest CT
Source: PLoS One. 2024 Feb 22;19(2):e0297390. doi: 10.1371/journal.pone.0297390 (PMC10883577; doi:10.1371/journal.pone.0297390)
Supplement: S2 Table — (DOCX) [file pone.0297390.s005.docx]

**S2 Table. Results of the Observer Performance Study for the Detection of Nodules with Lung-RADS 1.1 Categories 3 and 4**

| **Nodule** | **Nodule**  **character** | **Mean nodule diameter** | **Lung-RADS 1.1**  **Category** | **Reader 1**  **LDCT** | **Reader 1**  **ULDCT** | **Reader 2**  **LDCT** | **Reader 2**  **ULDCT** |
| --- | --- | --- | --- | --- | --- | --- | --- |
| Nodule 1 | Subsolid | 11.9mm (solid portion: 5.8mm) | 3 | 3 | 3 | 4A | 4A |
| Nodule 2 | Solid | 19.8mm | 4X | 4X | 4A | 4B | 4B |
| Nodule 3 | Subsolid | 21.8mm (solid portion: 10.5mm) | 4B | 4X | 3 | 4B | 4B |
| Nodule 4 | Solid | 7.6mm | 3 | Not detected | Not detected | 4A | 4A |
| Nodule 5 | Subsolid | 15.0mm (solid portion: 0.4mm) | 3 | 3 | 3 | Not detected | Not detected |
| Nodule 6 | Solid | 12.5mm | 4A | 3 | 3 | 4A | 4B |
| Nodule 7 | Solid | 11.9mm | 4A | 3 | 3 | 4A | 4B |
| Nodule 8 | Solid | 14.5mm | 4A | 4A | 4A | 4B | 4B |
| Nodule 9 | Solid | 14.0mm | 4A | 4A | 4A | 4B | 4B |
| Nodule 10 | Subsolid | 9.4mm (solid portion: 4.3mm) | 3 | 3 | Not detected | Not detected | Not detected |
| Nodule 11 | Solid | 6.3mm | 3 | Not detected | Not detected | 3 | 3 |
| Nodule 12 | Solid | 6.2mm | 3 | Not detected | Not detected | 4A | 3 |
| Nodule 13 | Solid | 6.0mm | 3 | Not detected | Not detected | 4B | 4B |
| Nodule 14 | Solid | 9.1mm | 4A | Not detected | Not detected | 4A | Not detected |
| Nodule 15 | Subsolid | 11.6mm (solid portion: 5.2mm) | 3 | 3 | 3 | 3 | 3 |
| Nodule 16 | Subsolid | 11.4mm (solid portion: 4.5mm) | 3 | 3 | 3 | 3 | 3 |
| Nodule 17 | Subsolid | 12.3mm (solid portion: 4.7mm) | 3 | 3 | 3 | 3 | 3 |

LDCT = low-dose chest computed tomography, ULDCT = ultralow-dose chest computed tomography.
