# Supplementary material for: Prospective evaluation of deep learning image reconstruction for Lung-RADS and automatic nodule volumetry on ultralow-dose chest CT
Source: PLoS One. 2024 Feb 22;19(2):e0297390. doi: 10.1371/journal.pone.0297390 (PMC10883577; doi:10.1371/journal.pone.0297390)
Supplement: S3 Table — (DOCX) [file pone.0297390.s006.docx]

**S3 Table. Nodule Detection Sensitivity of Lung VCAR and LuCAS Applied on Low-Dose Chest CT Reconstructed with ASIR-V_50_.**

|  | **_LDCT – ASIR-V50_** | |
| --- | --- | --- |
| **_Nodules_** | **_VCAR_** | **_LuCAS_** |
| **_Total_** | _72.1% (31/43)_ | _86.0% (37/43)_ |
| **_Solid_** | _77.8% (14/18)_ | _77.8% (14/18)_ |
| **_Subsolid_** | _14.3% (1/7)_ | _100% (7/7)_ |
| **_Calcified_** | _88.9% (16/18)_ | _88.9% (16/18)_ |

VCAR = volume computerized assisted reporting , LDCT = low-dose chest computed tomography, ASIR-V_50_ = adaptive statistical iterative reconstruction-V 50%
