# Supplementary material for: Prospective evaluation of deep learning image reconstruction for Lung-RADS and automatic nodule volumetry on ultralow-dose chest CT
Source: PLoS One. 2024 Feb 22;19(2):e0297390. doi: 10.1371/journal.pone.0297390 (PMC10883577; doi:10.1371/journal.pone.0297390)
Supplement: Attachment — Submitted filename: Response to Reveiwers.docx [file pone.0297390.s007.docx]

**Reviewer #1:** This study evaluated whether lung nodule detection, Lung-RADS classification, and volumetric nodule assessments were feasible with ULD chest CT scans with DLIR. Study included 40 patient cases reviewed by 2 observers with a commercialized assisted tool.
Authors concluded DLIR enabled comparable Lung-RADS and volumetric nodule assessments on ULDCT images to LDCT images.

**1. Study included assessment with a commercialized assisted tool (VCAR) in addition to observers' evaluation. Authors did not state the rationale of including the assessment using a software tool, and the reason why VCAR was introduced in this study is not clear.
If authors intended to use VCAR as a detection and measurement device, and compared nodule assessment performance by VCAR for LDCT and ULDCT scans with IR and DLIR, then the performance reliability of VCAR in terms of nodule detection and volume measurement need to be first assessed before drawing any conclusion based on measurements using that particular device. And yet, I could not find any description on that.
Authors should make it clear the role of VCAR assessment in this study and the way how its reliability was assessed.**

Response: Thank you for the keen comment.

Although there is a variety of software for automated detection and measurement of lung nodules in chest CT scans, the reason why we choose to use Lung VCAR is as follows:

First, among the various software for nodule detection and measurement for Chest CT scans, we thought using FDA-approved VCAR software from the same vendor (GE Healthcare) with our CT scanner could ensure consistency to this study. The purpose of our study is to compare the quality of DLIR compared to ASIR-V_50_ reconstruction and ULDCT to LDCT rather than evaluate the CAD software’s nodule detection ability and measurement accuracy.

Second, a previous study done by Lee et al. (Medicine 2020;99:23(e20543)) showed acceptable performance of Lung VCAR in nodule volume measurement with absolute percentage volume error around 10% in low-dose and standard-dose chest CT scans reconstructed with FBP and ASIR-V. Liang et al. (*AJR* 2017; 209:304–308) compared the volume of stable nodules in chest CT scans using two commercial software, Lung VCAR and SyngoVia. Lung VCAR showed a mean volume variation of 0.7% ± 18.6% (range, −39.3% to 83.3%) which was better than SyngoVia. These previous studies supported that Lung VCAR was reliable and became for our purpose.

Third, we initially assessed the performance of nodule detection by Lung VCAR for LDCT and ULDCT scans reconstructed with FBP, ASIR-V_50_, ASIR-V_100_ and DLIR and the nodule detection sensitivity showed consistent results, which was sufficient for analysis. The nodule detection sensitivities are listed in the table below.

|  | **_LDCT_** | | | | **_ULDCT_** | | | |
| --- | --- | --- | --- | --- | --- | --- | --- | --- |
| **_Nodules_** | **_FBP_** | **_ASIR-V 50_** | **_ASIR-V 100_** | **_DLIR-H_** | **_FBP_** | **_ASIR-V 50_** | **_ASIR-V 100_** | **_DLIR-H_** |
| _Total_ | _0.70 (30/43)_ | _0.72 (31/43)_ | _0.74 (32/43)_ | _0.72 (31/43)_ | _0.63 (27/43)_ | _0.65 (28/43)_ | _0.65 (28/43)_ | _0.67 (29/43)_ |
| _Solid_ | _0.78 (14/18)_ | _0.78 (14/18)_ | _0.78 (14/18)_ | _0.78 (14/18)_ | _0.67 (12/18)_ | _0.72 (13/18)_ | _0.72 (13/18)_ | _0.72 (13/18)_ |
| _Subsolid_ | _0.00 (0/7)_ | _0.14 (1/7)_ | _0.14 (1/7)_ | _0.14 (1/7)_ | _0.00 (0/7)_ | _0.00 (0/7)_ | _0.00 (0/7)_ | _0.14 (1/7)_ |
| _Calcified_ | _0.89 (16/18)_ | _0.89 (16/18)_ | _0.94 (17/18)_ | _0.89 (16/18)_ | _0.83 (15/18)_ | _0.83 (15/18)_ | _0.83 (15/18)_ | _0.83 (15/18)_ |

In response to your comment, we further thought about solutions to evaluate Lung VCAR’s reliability. Identifying a gold standard for nodule detection sensitivity and measurement was challenging since every software has limitations. Even in experienced radiologists, inter and intra-observer variability regarding nodule detection and measurement is inevitable.

We have compared VCAR with another commercially available software called LuCAS (Monitor Corporation, Seoul, Korea). LuCAS is a deep learning-based computer-aided diagnosis (DL-CAD) for nodule detection and segmentation in chest CT scans. The nodule detection sensitivity in LDCT with ASIR-V_50_ reconstruction showed no statistically significant difference between the two software (VCAR vs. LuCAS, 72% vs. 86%, p=.113). However, LuCAS performed better than VCAR in detecting subsolid nodules.

|  | **_LDCT – ASIR-V50_** | |
| --- | --- | --- |
| **_Nodules_** | **_VCAR_** | **_LuCAS_** |
| _Total_ | _0.72 (31/43)_ | _0.86 (37/43)_ |
| _Solid_ | _0.78 (14/18)_ | _0.78 (14/18)_ |
| _Subsolid_ | _0.14 (1/7)_ | _1.00 (7/7)_ |
| _Calcified_ | _0.89 (16/18)_ | _0.89 (16/18)_ |

The mean ± standard deviation of nodule volume difference between VCAR and LuCAS was 8.0 ± 78.9 mm^3^. The Bland-Altman plot below shows an acceptable measurement difference between the two software.

**2. In Fig. 5 (A) and (B), VCAR measurements already differ btw ASiR-LDCT and DLIR-LDCT quite a lot, which indicate VCAR's performance is not sufficiently reliable to distinguish between LDCT and ULDCT. Authors need to present the reliability range of VCAR's performance in order to justify its use in this study purpose.**

Response: Thank you for the comment. The answer to this comment will be presented together in questions 1 and 3.

**3. In Fig. 5 (B) and (C), volume measurement difference of case no. 17 exceeded 100%, which may result in a different patient management and represent a potential risk. And yet, authors did not mention that risk. Study results should be discussed in both positive and negative aspects.**
Response: Thank you for your keen comment.

In the waterfall charts of Figure 5 (B) and (D), one nodule’s volume difference exceeds 100% between ASIR-V_50_-LDCT and DLIR-LDCT and between ASIR-V_50_-LDCT and DLIR-ULDCT. The nodule was the smallest among the 26 nodules: a 4.5 mm-sized solid nodule. The Lung VCAR measured nodule volume was 25 mm^3^ in ASIR-V_50_-LD CT, 53 mm^3^ in DLIR-LDCT, and 60 mm^3^ in DLIR-ULDCT. Since the nodule is a small-sized solid nodule, Lung-RADS 1.1 category was 2 and despite the large percentage volume difference among ASIR-V_50_-LD CT, DLIR-LDCT, DLIR-ULDCT, the Lung-RADS 1.1 category did not change.

Previous studies also found larger measurement error in small-sized nodules by CAD and manual measurement by radiologists (AJR 2017; 209:304–308. Medicine 2020;99:23(e20543). Eur Radiol 2023;33:5568–5577.). This reaffirms that such inconsistencies aren't solely a limitation of the CAD software but are observed across different measurement techniques, particularly for smaller nodules.

In our study, Lung-RADS 1.1 category of 12 nodules with categories 3 and 4 stayed the same among ASIR-V50-LDCT, DLIR-LDCT, and DLIR-ULDCT. CAD software, including Lung VCAR, has limitations, but if used in the appropriate and specific situations, it would be helpful in clinical practice.

**4. M&M section states a total of 43 nodules were assessed with VCAR, but the number is 27 in Fig. 5. Needs to be clarified.**

Response: Thank you for pointing out the discrepancy. To clarify, we assessed the nodule detection sensitivity of VCAR for a total of 43 nodules. Out of theses, 26 nodules were consistently detected and their volume were measured by VCAR in all ASIR-V50-LDCT, DLIR-LDCT, and DLIR-ULDCT reconstruction images. Therefore, the comparisons in Lung-RADS 1.1 categorization and nodule volume difference were focused on these 26 nodules. Bland-Altman plots in Figure 4 and waterfall charts in Figure 5 show the 26 nodules’ volume difference. You are right about the number 27 in Figure 5d - it was an oversight on our part and has now been corrected to “26”. We apologize for any confusion this may have caused and appreciate your keen observation.

**5. Authors stated that there was no significant difference in the nodule detection sensitivity between the DLIR-LDCT and DLIR-ULDCT images. I assume authors used a chi-squire test for calculating the statistical significance, which is not clear to the reviewer. Please explain the way in detail.**

Response: We used the chi-square test for calculating the nodule detection sensitivity of each radiologist in DLIR-LDCT and DLIR-ULDCT scans. We added the detail in the statistical analysis as follows.

“ The chi-square test was conducted to compare the nodule detection sensitivities of each radiologist in DLIR-LDCT and DLIR-ULDCT.”

**6. For nodule detection study with observers, how were the gold standard nodules established ?**

Response: The gold standard nodules for nodule detection and Lung-RADS categorization was done by one board-certified radiologist with 7 years of experience in chest CT interpretation. We added the detail in the manuscript as follows.

“ One board-certified thoracic radiologist (***) reviewed 40 DLIR-LDCT scans thoroughly and detected nodules of Lung-RADS 1.1 category 3 or over for gold standard.”

**Reviewer #2:** LDCT reduces lung cancer mortality, but it needs annual/short-term follow-ups which deliver radiation exposures.a ULDCT is 6-10 times less dose than LDCT, and DLIR were shown to provide better image quality. This work attempts to evaluate classification and evaluation of the images, and the authors reached this goal using DLIR for hospital measurements. Given that it is informative on using DLIR for Ultra low dose chest CT, I think readers will benefit from this manuscript for their own practices. Before recommending for publication, I only have a few minor concerns on this manuscript.

**1. Introduction: "better image quality ... than the preexisting iterative reconstruction method (13-15)" suggests one single method but with multiple citations. Do the authors mean multiple methods?**

Response: We tried to cite three previous studies showing better image quality of deep-learning image reconstruction compared to the iterative reconstruction method. The citations (13-15) represent different studies, each comparing DLIR with a particular iterative reconstruction method. We apologize for any confusion caused by the wording.

**2. Study population: is the period of Feb-Jul 2020 chosen on purpose, because it overlaps with the onset of COVID-19 pandemic?**

Response: It was a coincidence. We started to plan this study in December 2019. IRB approval and clinical trial registration were completed in January 2020, before COVID-19 became a pandemic. In our country, the first COVID-19 patient occurred on January 1^st^, 2020. Due to the unexpected pandemic disease, the patient enrollment period took longer than we expected, but there was no significant impact on our study progress.

**3. Objective and subjective image quality assessment: the authors have four thoracic radiologists, with two for each task. Is the number too small for the assessment, if their assessment results diverge significantly?**

Response: Thank you for your concern. While we acknowledge that having only two radiologists for each task may seem limited, the reviewers were composed of board-certified thoracic radiologists with sufficient CT interpretation experience. Importantly, ICC showed excellent interobserver agreement for subjective assessment of image noise and diagnostic acceptability (0.82 and 0.86, respectively). Given the strong consistency in their evaluations, we believe their assessments provided meaningful and reliable results for this study.

**4. Statistical analysis:
(1) For the Bonferroni correction, why is 6 used?**

Response: Bonferroni correction compensates for the multiple comparisons we made in our analysis. For our study, we carried out multiple comparisons across 4 types of CT scans (ASIR-V50-LDCT, ASIR-V50-ULDCT, DLIR-LDCT, DLIR-ULDCT scans). The number of unique pairwise comparisons among these four scans is 6 (calculated as 4C2=6) Hence, we adjusted our significance level as follows:

Bonferroni-corrected p-value = original p-value / number of tests performed

0.008 = 0.05 / 6

**(2) For ICC thresholds are different across different studies, why did the authors choose 0.40 and 0.75? If this is a field-specific choice, can the authors provide citation to the choice?**

Response: Thank you for the comment. We do agree with the reviewer’s opinion that ICC thresholds differ across studies, therefore reference is necessary. We cited the article written by Domenic V. Cicchetti written in 1994 and added the reference no. 18 for the ICC thresholds in the manuscript.

**(3) Does Fleiss' kappa offer a different result, if offered?**

Response: Thank you for your keen question. Prior to writing this manuscript, we had statistical consultation. ICC was recommended for the interobserver agreement in subjective image quality assessment of the CT scans, and kappa was not recommended. Since the subjective image quality assessment was a scoring task (ordinal variable), kappa, which is for the nominal variable, was not applicable. Cohen’s Kappa was recommended for the Lung-RADS 1.1 categorization agreement between two radiologists, and Fleiss’ Kappa, which is for interobserver agreement for 3 or more raters, was not applicable.

**5. Table 1: what are the two values in the brackets of 21 (53) and 19 (47)?**

Response: The data are the number of patients with percentages in parentheses. We indicated in the footnotes as follows.

^“ †^ Data are number of patients with percentage in parentheses.”

**6. Table 2: * - could the mention of Bonferroni here be more clear for the adopted significance level?**

Response: Thank you for the comment. We amended our footnotes and mentioned Bonferroni as follows.

“ * Bonferroni corrected p value < .008 indicated a statistically significant difference.”

**7. Nodule detectability and Lung-RADS categorization by VCAR: given that there is no significant difference between the two sets of images, would the authors like to mention that DLIR-ULDCT should be preferred given its ultra low dose?**

Response: We appreciate your observation. There was no significant difference in the nodule detection sensitivity of VCAR between the DLIR-LDCT and DLIR-ULDCT images (72.1% and 67.4%, P=.64). Lung-RADS 1.1 categorization of 12 nodules of category 3, 4 nodules by VCAR were exactly the same. Therefore, DLIR-ULDCT is a scan comparable to DLIR-LDCT. However, in previous studies, it is known that the measurement accuracy of VCAR is poor when the size of the nodule is small, so the accuracy may be low for category 2 nodules. In actual clinical practice, nodule detection CAD for chest CT scans is not popularized, and nodule detection and risk assessment are usually performed by a radiologist. In our observer study, radiologists’ nodule detection sensitivities did not significantly differ between DLIR-LDCT and DLIR-ULDCT (70.6%-88.2% vs. 64.7%-82.4%, P=.57). In terms of Lung-RADS 1.1 categories, the two radiologists did not detect or down-categorized three and two nodules each in DLIR-ULDCT compared to in DLIR-LDCT. Therefore, we advocate DLIR-ULDCT primarily for follow-up scans for monitoring pulmonary nodules rather than initial assessments. We have incorporated this in the conclusion.

**8. Figure 3: can the authors highlight certain features that were not visible with ASIR-V50 but evident with DLIR?**

Response: DLIR showed superior lesion conspicuity and small vessel visibility in the lung window setting compared to the ASIR-V_50_ reconstruction algorithm in both low-dose and ultralow-dose chest CT scans. Additionally, in the mediastinal window setting, mediastinal lesions and anatomical structures were more clearly depicted in DLIR compared to ASIR-V_50_ reconstruction algorithm in both low-dose and ultralow-dose chest CT scans. We have added some figures in Figure 3 to exemplify these points.
