# Supplementary material for: Prospective evaluation of deep learning image reconstruction for Lung-RADS and automatic nodule volumetry on ultralow-dose chest CT
Source: PLoS One. 2024 Feb 22;19(2):e0297390. doi: 10.1371/journal.pone.0297390 (PMC10883577; doi:10.1371/journal.pone.0297390)
Supplement: Attachment — Submitted filename: Response to reveiwer_231219.docx [file pone.0297390.s008.docx]

**Response to the Reviewer**

**1. There are significant controversies surrounding the actual risk of low-dose radiation exposure for adults. For instance, a recent study has indicated that LDCT does not induce DNA damage (source:** [**https://doi.org/10.1148/radiol.2020190389**](https://doi.org/10.1148/radiol.2020190389)**). Whether LDCT entails "non-negligible radiation exposure" remains unclear. The description requires modification.**

**Response:** Thank you for your comment. The study by Sakane et al. proved that a single low-dose CT with a median effective dose of 1.5mSv does not damage human DNA in peripheral blood lymphocytes. While these findings are innovative and potentially impactful, they do not encompass patient groups who undergo annual or more frequent low-dose CT scans.

In clinical practice, repeated low-dose CT scans are common due to various conditions, making difficult to ignore the effects of cumulative radiation exposure in these cases. Therefore, clinicians and radiologists continue to make efforts to minimize radiation exposure in patients.

We amended the description as below:

A recent study by Sakane et al. reported no damage of human DNA from a single low-dose CT scan [6]. However, lung cancer CT screening and pulmonary disease evaluations may often involve the frequent use of follow-up CT scans [7], and the safety of the cumulative radiation exposure remains underexplored.

**2. It is unclear how to determine the sample size (n=40) for this prospective study. Please provide clarification.**

**Response:** We assumed that deep-learning image reconstruction in ultra low-dose CT scan group will obtain 90% of satisfactory subjective overall quality and iterative reconstruction in ultra low-dose CT scan group will obtain 65% of satisfactory subjective overall quality. Applying the alpha error =0.05, and beta error =0.20 (power=0.80), the calculated total sample size was 40.

**3. Please specify the number of patients excluded based on each exclusion criterion.**

**Response:** Since this study was a prospective cross-sectional study, the patient enrollment was done by asking patients who met the inclusion criteria and did not meet the exclusion criteria their willingness to participate in this study and obtaining their consent at a pulmonology outpatient clinic center about. Therefore, the total number of the all potential participants who met the inclusion criteria or met the exclusion criteria was not separately recorded.

**4. Please include information about the noise index for both LDCT and ULDCT protocols if the authors utilized automated tube current modulation. This crucial information should be presented in the main text rather than the supporting text.**

**Response:** Thank you for the comment. The noise index for LDCT was 28 and for ULDCT was 33. We relocated the S1 text in the main manuscript and added the noise index as follows:

Consecutive full-inspiratory thoracic LDCT scans at 120 kVp and ULDCT at 100 kVp were acquired in all patients using a single CT machine (Revolution CT; GE Healthcare, Waukesha, WI, USA) and the scanning parameters were as follows: tube voltage, 120 kVp with a mAs of 25 for LDCT, 100 kVp with a mAs of 5 for ULDCT; automatic tube current modulation; gantry rotation time, 280 ms; detector configuration, 128 × 0.625 mm; beam pitch, 1.53; matrix, 512x512; reconstruction increment and section thickness, 1.25 mm; noise index for LDCT, 28 and noise index for ULDCT, 33.

**5. Why did the authors need to treat S2, S3, and S4 texts as supporting text? This important information should be included in the main text, unless there are reasonable constraints such as word count limitations.**

**Response:** Thank you for the comment. As you suggested we relocated the S2, S3, S4 text within the main manuscript.

**6. The McNemar test is recommended for intraindividual comparisons of nodule detection sensitivity, rather than the Chi-square test.**

**Response:** Thank you for the keen comment. As your advice, we reconducted McNemar test to compare the nodule detection sensitivities of each radiologist and VCAR between DLIR-LDCT and DLIR-ULDCT and to compare the nodule detection sensitivity of VCAR and LuCAS. Each p value number itself has changed, but the overall statistical significance has not changed. The changes were as follows:

Radiologists’ nodule detection sensitivities between DLIR-LDCT and DLIR-ULDCT, P=.57 → P=1

VCAR’s nodule detection sensitivity between DLIR-ULDCT and ASIR-V50-ULDCT, P=.99 → P=1

VCAR’s nodule detection sensitivity between DLIR-LDCT and DLIR-ULDCT, P=.64 → P=.5

Nodule detection sensitivity between VCAR and LuCAS, P=.113 → P=.18

Subsolid nodule detection sensitivity between VCAR and LuCAS, P=.002 → P=.03

**7. One of the major limitations in this study is that the authors used a vendor-specific DLIR algorithm, and as a result, the applicability to other DLIR algorithms provided by different vendors is not discussed. Recent investigations employed a different vendor-specific DLR for ULDCT of the lung to improve image quality (doi: 10.1016/j.acra.2022.04.025 and doi: 10.2214/AJR.19.22680.). Please provide a concise discussion of whether your results align with these prior investigations to offer readers the wide applicability of study findings.**

**Response:** We appreciate the comments. As you suggested, we added previous studies of DLIR in discussion section as follows:

There are previous studies of a vendor-specific and vendor-agnostic DLIR to create high quality images of ULDCT [26, 27]. In a study by Goto et al., ULDCT with vendor-specific DLIR (AiCE, Canon Medical Systems) showed better nodule CNR and comparable subjective image quality assessment (noise in air, noise in soft-tissue, streak artifact, texture fineness, and overall quality) compared to LDCT with iterative reconstruction in the phantom study [26]. In a subjective evaluation, ULDCT with DLIR of 14 nonobese patients achieved a significantly higher preference by radiologists compared to ULDCT with iterative reconstruction [26]. Hata et al. proved a vendor-agnostic DLIR for noise reduction of ULDCT showed better subjective image quality compared to the ULDCT with iterative reconstruction [27]. Also, nodule detection rate of radiologists improved in ULDCT with DLIR compared to ULDCT with iterative reconstruction [27]. These previous studies and ours both align with the same result that ULDCT with DLIR increases objective and subjective image quality compared to iterative reconstruction algorithms and provides image quality comparable to that of LDCT.

**8. The study findings may be applicable for pediatric CT, where radiation exposure is of concern in comparison to adults who require lung cancer screening. Discussing the applicability to low-dose pediatric CT by citing the relevant articles (such as doi: 10.1148/rg.2021210105. and doi: 10.1186/s12880-021-00677-2.) would further emphasize the value of the authors' findings.**

**Response:** Dose reduction in pediatric CT using DLIR and ULDCT is a very novel and influential idea and thank you for suggesting a perspective that we hadn’t thought of before. We added this to discussion section as follows:

There have been concerns regarding the increased radiation exposure from medical imaging of pediatric populations [28]. Efforts to reduce the radiation dose in pediatric CT imaging continued steadily and dose reduction strategies in CT imaging is expected to dramatically reduce the adverse effects of radiation exposure such as radiation-induced cancers [28, 29]. One of the proven dose reduction strategies is DLIR which can minimize image noise while preserving the adequate resolution of the CT scans [29, 30]. In a study by Yoon et al., in pediatric non-contrast and contrast-enhanced chest CT (CTDIvol, 1.3 ± 0.5 mGy; DLP 49.0 ± 26.3 mGy × cm; effective dose, 2.2 ± 3.2 mSv), DLIR showed significant noise reduction with increased SNR and CNR and showed better subjective overall image quality and noise compared to iterative reconstruction [30]. This result is comparable to our results and application of DLIR is expected to substantially reduce the radiation dose while maintaining the appropriate image quality for diagnosis in pediatric chest CT scans.
